# The coming wave of change: ICD-10

**DOI:** 10.4103/2153-3539.74183

**Published:** 2010-12-23

**Authors:** Ji Yeon Kim, Bruce A. Beckwith

**Affiliations:** Department of Pathology, Massachusetts General Hospital, 55 Fruit Street, Boston, Massachusetts 02114; 1 North Shore Medical Center Salem Hospital, 81 Highland Avenue, Salem, Massachusetts 01970

Statistics on diseases and causes of death have been important to governments for a wide variety of reasons, including tracking changes in population health, guiding resource allocation, setting research agendas, among others. In 1893, the World Health Organization (WHO) established the International List of Causes of Death,[1] the precursor to the International Classification of Diseases (ICD), as a standard coding system for reporting mortality data, an important advance for longitudinal and comparative studies. The ninth revision of ICD was expanded in the United States to more precisely capture morbidity data and to also include codes for surgical procedures, and is known as ICD-9-CM (Clinical Modification). In 1983, ICD-9-CM began to be used for reimbursement purposes, which remains one of its key functions today.

Currently, over 99 percent of Medicare Part A claims and over 96 percent of Part B claims are received electronically.[2] The Health Insurance Portability and Accountability Act of 1996 (HIPAA) has set standards for healthcare transactions, among which are ICD-9-CM, Current Procedural Terminology (CPT) and the Health Care Financing Administration Common Procedure Coding System (HCPCS), National Drug Codes (NDC) and the Code on Dental Procedures and Nomenclature.

The tenth revision of ICD[3] was approved in 1990, and it has been widely adopted throughout the world for mortality reporting, including in the United Kingdom (1995), France (1997), Australia (1998), United States (1999), Germany (2000), and Canada (2001). In addition, most industrialized countries have adopted morbidity classification systems based on ICD-10, such as Canada, which uses ICD-10-CA. There are a number of reasons that make moving away from ICD-9-CM desirable - it is outdated, has a relatively small number of codes, lacks the specificity and detail of newer coding systems and is essentially out of space to add new codes. In addition, the lack of specificity makes it difficult to use ICD-9-CM to support biosurveillance or for pay for performance arrangements. ICD-10-CM, on the other hand, allows much greater specificity in coding, which should enable a wide range of financial, quality and disease monitoring efforts to be improved.

The Department of Health and Human Services issued a final rule on January 16, 2009,[4] adopting ICD-10-CM and ICD-10-PCS as the standard code sets for use in reporting diagnoses and inpatient hospital procedures, respectively, in healthcare transactions. The compliance date has been set as October 1, 2013, to coincide with the annual Medicare inpatient prospective payment system updates. A second related rule changes the electronic transaction standard from version 4010/4010A1 to version 5010, which will be necessary to accommodate the ICD-10-CM/PCS codes. This transition is scheduled for January 1, 2012, well in advance of the target date for implementing the new coding system.

ICD-10 has a significantly different structure from ICD-9, which makes it possible to encode more information, such as laterality where relevant, and reflects modern medical terminology.[5] ICD-10-CM diagnoses codes developed by the Centers for Disease Control and Prevention (CDC) are similar but not the same in format as ICD-9-CM. In ICD-9-CM, diagnoses codes can be three to five digits long, with the first digit being alpha or numeric, and the rest being numeric characters. In contrast, ICD-10-CM diagnoses codes can be three to seven digits, with the first digit being alpha, second digit being numeric, and digits three through seven being either alpha or numeric. Similarly, for procedures performed in inpatient hospital settings, the three to four numeric digits currently used in the ICD-9-CM procedure coding system will be replaced by seven alpha or numeric digits in the new ICD-10-PCS procedure classification system developed by the Centers for Medicare and Medicaid Services (CMS). ICD-10-CM/PCS will not affect the use of Current Procedural Terminology (CPT) codes on Medicare claims for outpatient procedures, however, as CPT use will continue.

As a result of these changes, ICD-10-CM/PCS contains many more codes than ICD-9-CM (roughly 155,000 vs. 17,000), making it difficult to directly translate codes from one system to the other. Because of these differences, CMS and the CDC have created the General Equivalence Mappings (GEMs) to act as translation dictionaries. These are public domain reference mappings that are meant to serve in the specific capacity of converting databases, linking data in long-term clinical trials, developing application-specific mappings and analyzing data collected before and after the transition to ICD-10-CM/PCS.

An important consideration for the future, however, is that clinicians will have to provide much more detailed documentation to ensure correct coding using ICD-10-CM/PCS; failure to do so will likely result in increased workload for case managers and health record professionals, potentially delaying reimbursements and discharge planning for post-acute care, even after a “successful” conversion to the new ICD-10-CM/PCS coding system.[6] During this learning period, production of diagnostic interpretations and results may be delayed with potential impact on patient care, and the rate of missing or invalid diagnosis codes will likely increase in the near future.

Clinical laboratories also rely heavily on valid diagnosis codes on laboratory test orders from physicians as part of the billing process and in some cases to establish medical necessity for diagnostic testing.[7] LISs typically collect ICD codes from ordering providers on paper or electronic requisition forms, store them in the LIS and then transfer them to a number of other information systems by electronic interfaces. Almost all laboratories will need to upgrade and test external interfaces with ordering providers as well as payers to whom claims are sent, and this must be coordinated prior to implementation. Internal interfaces must be identified and modified as well.

Identifying and tracing the final destination of the data may be particularly complex for laboratories. Interfaced systems may include not only the hospital information system (HIS), but also various data repositories, government or public health agencies, and electronic medical record systems (EMRs), each of which may also interact with others. At a national level, aggregated coded data are used for multiple purposes, including the Centers for Medicare and Medicaid Services (CMS) Hospital Compare web page (http://www.hospitalcompare.hhs.gov/), National Center for Health Statistics (http://www.cdc.gov/nchs/) web page, and individual state web pages.

Given the interdependence of these data systems and the wide variety of ways that ICD codes may be used, such as in medical necessity verification policies, pay for performance arrangements, among others, the maintenance and updating of codes is not an insignificant task. It will be important to manage the conversion process such that upgrades to systems, whether by vendors, payers, or data warehouses, are coordinated, rather than being allowed to occur independently. This should be done to avoid a “domino” effect, where an uncoordinated upgrade is done and the interface fails due to the receiving system being unable to process the new transaction format.

Monetary costs of conversion are expected to be high. National estimated costs for the United States conversion from ICD-9-CM to ICD-10-CM/PCS range from several hundred million to several billion dollars, including costs for training, system upgrades, productivity losses, and contract re-negotiations. One large national reference laboratory has estimated its own cost for implementation of ICD-10-CM/PCS at $40 million, including information technology and training expenses.[7] A study by the Rand Corporation in 2004 estimated one-time conversion costs between $425 million and $1.15 billion, as well as an additional $5 million to $40 million a year in lost productivity; estimated benefits were between $700 million and $7.7 billion for more accurate payments, fewer rejections, fewer fraudulent claims, better understanding of new procedures and improved disease management.[8]

The United States Department of Health and Human Services used data from a variety of sources to perform its own cost-benefit analysis of the ICD-10 conversion process, estimating the total costs of implementation would be about $1.64 billion (range $849 million to nearly $3.05 billion).[9] In this analysis, the costs would be quantitatively matched by overall societal benefits by the year 2018, in terms of more accurate claims, fewer rejected claims, fewer improper claims, better understanding of new procedures, improved disease management, better understanding of health conditions and outcomes, and harmonization of disease monitoring and reporting worldwide, although the latter two outcomes were not included in the dollar estimates.[9]

For clinical laboratories, costs will vary by the service model [Table 1]. Each laboratory should begin by performing its own business process analysis to identify needed changes, including reviewing how diagnostic codes are being documented and billing procedures. Depending upon their size and complexity, laboratories may consider assigning a dedicated administrator and budgeting several weeks to months for this task. Education and training of staff, particularly billing staff, on the new codes will be required, whether by self-paced online courses or by on-site training. Clients will also need to be educated on the transition and process changes. Paper or web-based requisitions and manuals that include ICD codes should be updated. Electronic changes will include the LIS, laboratory billing systems, middleware, electronic ordering system, and medical necessity checking software. In addition, interfaces will need to be updated with the hospital information system (HIS), electronic medical record (EMR), research databases, and any other relevant downstream systems. These electronic changes will also need to be tested and validated.

**Table 1:** List of possible costs associated with switching to ICD-10-CM/PCS for laboratories

Cost categories	Detail
Business process analysis	Review current contracts
	Review how diagnostic codes are being documented
	Review billing procedures
Education and training	Education and training of staff, especially billing staff
	Education of clients regarding the transition and process changes
	Increased documentation
Paper changes	Changes to paper requisitions and manuals that include ICD codes
Electronic system changes	Changes to laboratory information systems
	Changes to laboratory billing systems
	Changes to middleware and electronic ordering system
	Changes to medical necessity checking software
Interface changes	Review current interfaces with other information systems
	Changes to interfaces between LIS and billing and/or HIS systems
	Changes to interfaces between LIS and client EMR
Testing/Validation	Testing and validation of software and interface changes

Laboratories should be investigating the steps needed to accommodate ICD-10-CM/PCS in their LIS systems and interfaces now, and be prepared to add and test this new functionality in advance of the target date. Coupled with the recent governmental monetary incentives for physician groups to adopt EMRs, there will likely be significant pressure on laboratories to implement ordering and result interfaces which are able to use ICD-10-CM/PCS with their clients, and this will stretch internal LIS resources thin. Given the magnitude of this change and the number of entities and systems involved, it is possible that the target implementation date will be moved back (it was already moved back two years from the 2011 date initially proposed), but prudence suggests that early planning is wise.

**Competing interests:** The authors declare that they have no competing interests.
